# Cost-effectiveness analysis of a telemedicine network for acute neurological care in northeast Germany (ANNOTeM)

**DOI:** 10.3389/fdgth.2026.1749113

**Published:** 2026-04-17

**Authors:** Janina Behrens, Tobias Kurth, Malgorzata Kotlarz-Böttcher, Anselm Angermaier, Sarah Theen, Rohat Geran, Heinrich J. Audebert

**Affiliations:** 1 Department of Neurology, Charité – Universitätsmedizin Berlin, Corporate Member of Freie Universität Berlin and Humboldt-Universität zu Berlin, Berlin, Germany; 2 Center for Stroke Research Berlin, Charité – Universitätsmedizin Berlin, Corporate Member of Freie Universität Berlin and Humboldt-Universität zu Berlin, Berlin, Germany; 3 Institute of Public Health, Charité – Universitätsmedizin Berlin, Berlin, Germany; 4 Department of Neurology, University Hospital Greifswald, Greifswald, Germany; 5 Department of Psychiatry, Psychosomatics and Psychotherapy, Protestant Hospital Bethanien, Greifswald, Germany; 6 Independent Trust Office of University Medicine Greifswald, Greifswald, Germany

**Keywords:** cost-effectiveness, digital health, quality improvement, telemedicine, telestroke

## Abstract

**Background:**

Expert neurological care in rural areas remains a major challenge and contributes to disparities in outcomes after acute neurological emergencies. To address this gap, the ANNOTeM project established a comprehensive, digitally enabled “hub-and-spoke” telemedicine network connecting academic neurology centers with regional hospitals in northeast Germany, providing 24/7 remote expertise, standardized operating procedures, and digital quality management.

**Methods:**

This prospective pre and post implementation study used statutory health insurance claims to compare patient outcomes and costs for acute neurological emergencies across 11 ANNOTeM network hospitals vs. 11 matched non-network hospitals (all hospitals were localized in rural regions). The analysis included all consecutively hospitalized adults with ICD-10 coded acute neurological disorders. The primary clinical endpoint was the composite of 90-day mortality, new need for outpatient or nursing home care. Health economic evaluation included direct medical, non-medical, and indirect costs from the insurer's perspective.

**Results:**

Following network implementation, the rate of the primary outcome decreased in ANNOTeM hospitals (33.8% vs. 35.9%; unadjusted absolute difference: −2.1%; adjusted absolute difference: −3.2%; aHR 0.89, 95% CI: 0.79–0.99), with no improvement in control hospitals (40.7% vs. 42.5%; aHR 1.04, 95% CI: 0.85–1.15). Mean 90-day total costs per patient rose modestly from €11,938 to €12,252 (+2.6%, non-significant). Non-network hospitals showed a similar non-significant cost increase. The cost per avoided adverse composite outcome was €14,968 (unadjusted).

**Conclusion:**

Implementing a digitally integrated teleneurology network was associated with improved patient outcomes without substantial increases in per-patient costs. These results support the economic sustainability and transformative potential of innovative, network-based telemedicine systems for acute neurological care in underserved, rural regions.

## 1 Introduction

Acute neurological diseases, like stroke, status epilepticus, meningitis/encephalitis, or acute spinal cord injury, are among the main causes of long-term disability and mortality (1–5). Clinical outcomes of patients with these conditions depend on the promptness and accuracy of diagnosis and treatment. Evidence-based treatments, such as recanalizing therapies in stroke, specialized stroke unit care, or early decompressive surgery in spinal cord injury, have been shown to improve patient outcomes significantly (6–9).

However, in rural and structurally underserved areas, timely access to neurologically specialized acute care remains limited. For example, such structural deficits are very pronounced in Northeast Germany, part of the former German Democratic Republic, where low population density and a high average age pose challenges for health care in general and, in particular, for the adequate management of age-related diseases such as stroke. Shortages of medical staff and specialized hospital facilities make it even harder to provide timely neurological care in all regions (10, 11). As a result, patients living in such regions in Germany—and similarly in other rural parts of Europe and the USA—face an increased risk of delayed or inadequate care, leading to long-term dependency, institutionalization, and higher healthcare costs (12–16).

In response to these challenges, telemedicine has emerged as a powerful organizational tool to improve access to neurological expertise across geographically dispersed care settings. For example, telestroke models have demonstrated effectiveness and cost-efficiency in improving access to thrombolysis and specialized stroke care in underserved regions (15, 17–19). The ANNOTeM network (20) was designed as a comprehensive, regionally integrated system connecting three academic neurological centers with regional hospitals, adding dedicated facilities for neurological emergencies and comprehensive quality management to existing onsite or remote neurological consultation services. At the time of this analysis, 11 network hospitals in Northeast Germany (Mecklenburg-Western Pomerania, Brandenburg, and Saxony-Anhalt) were included. The ANNOTeM network also covers a broader range of acute neurological emergencies besides stroke, like coma, status epilepticus, meningitis/encephalitis, and acute spinal cord injury (20, 21).

Despite a growing body of evidence supporting the clinical benefits of telemedicine in acute neurology (22, 23), robust health economic evaluations of telemedicine-enhanced systems of care remain essential to inform policy decision-makers and guide broader implementation.

Detailed analyses of clinical outcomes of the ANNOTeM managed care system, including the composite endpoint of death, new need for ambulatory or nursing home care, and subgroup analyses, have been reported previously by Erdur et al. (21) in a separate publication using the identical network and study design. Building on these findings, this study aims to provide a comprehensive health economic evaluation of the ANNOTeM teleneurology network, using insurance claims data and standardized costing methodology.

## 2 Materials and methods

### 2.1 Study design and intervention

This multicenter, controlled intervention study was carried out in 11 acute care hospitals in Northeast Germany, matched for structure and region, all participating in a network led by three university hospitals (Charité—Universitätsmedizin Berlin, Universitätsmedizin Greifswald, and BG Unfallkrankenhaus Berlin). The study was designed as a comparison between two periods with patients admitted during a pre-implementation phase (October 1st, 2014 to March 31st, 2017) and a post-implementation phase (November 1st, 2017 to July 31st, 2020) under the assumption that patient characteristics would not change between the two periods when adjusted for age and sex. The post-implementation period had to be shortened by 5.5 months (until February 15th, 2020) because the assumption of unchanged patient characteristics no longer held true, as the avoidance of hospital admission during the early phase of the COVID-19 pandemic led to increased average stroke severity among hospitalized patients. The protocol amendment had been approved by the Ethics Committee (protocol amendment to EA1/078/10) and the funder before any data from health insurances were exported for statistical analyses. Clinical outcomes, including mortality, new need for ambulatory or nursing home care, and institutionalization within 90 days, were predefined and analyzed in detail in a separate outcome paper by Erdur et al. In the present manuscript, these outcome data are used as contextual background, while the primary focus lies on the analysis of costs and cost-effectiveness based on statutory health insurance claims (21).

For both phases, 11 hospitals, selected for their similarity in geographic region, case volume, and size, served as structurally matched controls. None of the control hospitals were involved in the ANNOTeM network or other comprehensive telestroke services during the time of the study.

The intervention included the setup of new dedicated units for neurological emergencies (TeleNeuro-Units) in each hospital, a 24/7 teleconsultation service provided by offering board-certified neurologist standards, standardized operating procedures for diagnosis and treatment of acute neurological emergencies, a structured quality management system, and repetitive multiprofessional training. In each participating hospital, a TeleNeuro-Unit was established as a defined area on an acute ward or stroke unit with monitored beds and routine access to brain imaging. These units were equipped with a fixed or mobile high-resolution remote control video and audio system connected to a secure telemedicine platform, enabling real-time consultations with neurologists at the hub centers. Local physicians and nurses provided bedside care, vital sign monitoring, and diagnostic work-up, while remote neurologists at the hubs contributed specialist expertise, guideline-based diagnostic and treatment protocols, and continuous quality management.

The ANNOTeM network was organized as a structured hub-and-spoke system linking three academic neurology centers (hubs) with 11 rural acute care hospitals (spokes). Rural hospitals were equipped with jaccess to network-wide treatment algorithms for acute neurological emergencies. Whenever an acute neurological case was admitted, the local team could initiate a teleconsultation 24/7 via a dedicated hotline that directly connected to the on-call offering board-certified neurologist standards at one of the academic centers. The hub neurologist reviewed clinical information and brain imaging in real time, provided immediate diagnostic and therapeutic recommendations (e.g., indication for thrombolysis, need for transfer for mechanical thrombectomy or neurosurgery), and documented these in a shared digital quality management platform. For patients requiring higher-level interventions, the hub neurologist coordinated secondary transfer to a suitable comprehensive center, while patients who could be safely managed locally remained in the rural hospital under continued tele-neurological supervision. In addition, regular multiprofessional trainings and feedback conferences between hub and spoke sites were implemented to harmonize treatment pathways, discuss challenging cases, and continuously update local teams on guideline-concordant acute neurological care.

### 2.2 Study population

All included patients had a hospital main diagnosis code of an acute neurological emergency, according to ICD-10 codes: I60–I64, G41, R40, A82.0–2, A85–87, G00–03, G06–09, S14, S24, S34, G82). Other inclusion criteria were age over 18 years of age, and insurance at one of the following statutory health insurance providers, which cover approximately 60%–70% of patients in the involved hospitals: AOK Nordost, BARMER, or Techniker Krankenkasse. In contrast to the evaluation of the clinical outcomes across 4 health insurance providers (AOK Nordost, AOK Sachsen-Anhalt, BARMER, Techniker Krankenkasse) patients insured at the AOK Sachsen-Anhalt had to be excluded from the health economics analysis because cost data were not provided.

### 2.3 Cost assessment

Costs were assessed from the statutory health insurance perspective, including the social long-term care insurance. We included direct medical costs (inpatient care, rehabilitation, outpatient care, pharmaceuticals, medical aids and devices, nursing and long-term care), direct non-medical costs (home help, patient transport), and indirect costs (productivity losses due to sick pay). In line with the original evaluation report, we additionally included the implementation-related costs of the ANNOTeM teleneurology network (teleconsultation services, central and regional network coordination, and training) as an intervention-specific per-case lump sum of €715.33 per acute neurological case, which was added to the claims-based costs in the post-implementation period (yielding mean total 90-day costs of €12,252.12 vs. €11,937.81 and incremental costs of €314.32 per insured person in network hospitals). All costs were standardized to 2021 values and adjusted using a cumulative general consumer price inflation of 3.7% for the period 2018–2021.

### 2.4 Outcome measures and statistical analysis

The primary endpoint was a composite of death, first-time need for long-term nursing care, and first-time institutionalization within 90 days after hospital admission. Cost effectiveness was evaluated by estimating the incremental cost effectiveness ratio (ICER), defined as the difference in total costs between the intervention and control groups divided by the difference in the occurrence of the composite endpoint.

Statistical analysis included estimation of means with corresponding 95% confidence intervals, and subgroup analyses stratified by age, sex, diagnosis, and baseline care status. Sensitivity analyses assessed the robustness of findings under varying assumptions and included the impact of the COVID-19 pandemic on care utilization patterns.

### 2.5 Ethics and data protection

This study was approved by the institutional ethics committee at Charité – Universitätsmedizin Berlin (No. EA1/078/10), and by the ethics committees of the chambers of physicians in Brandenburg [AS 90(bB)/2018] and Mecklenburg-Western Pomerania (BB 057/18). Data protection approval was granted by the TMF Technology and Methods Platform for Networked Medical Research and by the data protection officers of the Charité and all participating centers. All analyses were performed on double-pseudonymized health insurance claims data in accordance with German legal requirements (§75 SGB X). No patient identifiers were accessible to the research team; informed consent was not required, as only routinely collected, secondary insurance data were used, in accordance with German law and with approval from the relevant authorities.

## 3 Results

In the clinical outcomes analysis (21), the primary outcome occurred in 479/1,418 (33.8%) patients in the post-implementation and in 829/2,306 (35.9%) patients in the pre-implementation period, corresponding to an unadjusted absolute difference of −2.1%. The adjusted absolute difference was −3.2% and the adjusted hazard ratio (aHR) 0.89 (95% confidence interval [CI]: 0.79–0.99, *p* = 0.04). No improvement was seen in non-participating hospitals in the post- [757/1,781 (42.5%)] vs. the pre-implementation [869/2,135 (41%)] period (aHR 1.04; 95% CI: 0.95–1.15).

A total of 7,302 patients were included in the health economics analysis: 2,113 in the pre-implementation period group and 1,273 in the post-implementation group of the ANNOTeM network hospitals; and 2,135 in the pre-implementation group and 1,781 in the post-implementation period of non-network hospitals. Baseline characteristics, including age, sex, and preexisting care needs, are summarized in Table 1. The most common diagnosis was cerebral infarction (ICD-10: I63), accounting for approximately 90% of all cases. The full diagnostic distribution is presented in Table 1.

**Table 1:** Patient characteristics in ANNOTeM network hospitals.

Variable	Pre-implementation period *n* (%)	Post-implementation period *n* (%)
Mean age (years, SD)	75.6 (SD 11.0)	76.4 (SD 10.1)
Female (%)	1,177 (55.7%)	693 (54.5%)
With care needs (%)	790 (37.4%)	522 (41.0%)
With nursing home care (%)	195 (9.2%)	134 (10.5%)
Diagnosis (ICD) of ANNOTeM network hospitals	Pre-implementation period *n* (%)	Post-implementation period *n* (%)
Cerebral infarction (I63)	1,936 (91.7%)	1,141 (89.7%)
Intracerebral hemorrhage (I61)	55 (2.6%)	40 (3.1%)
Subarachnoid hemorrhage (I60)	8 (0.4%)	4 (0.3%)
Unspecified stroke (I64)	53 (2.5%)	35 (2.7%)
Spinal cord syndromes (S14/S24/…)	12 (0.6%)	9 (0.7%)
Meningoencephalitis (A85/A86/A87/G00/…)	19 (0.9%)	21 (1.6%)
Unclear consciousness/Status (R40/G41)	18 (0.9%)	17 (1.3%)
Others	12 (0.6%)	6 (0.5%)
Total	2.113 (100%)	1.273 (100%)

Data refer to baseline characteristics at hospital admission. “With care needs” refers to patients with officially recognized dependency under the German long-term care insurance system, including those receiving home-based care or residing in nursing homes. Distribution of main ICD-10 diagnoses among patients included in the cost analysis cohort (i.e., those with complete cost data) in ANNOTeM network hospitals before (pre-implementation period) and after network implementation (pos-implementation per). Percentages refer to the respective group. SD, standard deviation.

No significant cost differences were observed between female and male patients in either group, with mean costs in the intervention group of €12,286 for women (95% CI: €11,692–€12,880) and €12,369 men (95% CI: €11,713–€13,024), and in the post-implementation group of €12,101 for women (95% CI: €11,451–€12,750) and €12,163 for men (95% CI: €11,610–€12,716). Patients diagnosed with stroke incurred higher costs (€12,553 in the intervention group) compared to those with other acute neurological diagnoses (€9,670). Among patients with preexisting care needs, average costs in the intervention group were €12,213 (95% CI: €11,451–€13,053) compared to €10,492 (95% CI: €10,019–€10,981) in the post-implementation period. In subgroup analyses stratified by baseline care status, patients with and without pre-existing care needs were analyzed separately regarding mean 90-day costs and cost changes between the pre- and post-implementation periods. The results did not show a statistically significant interaction between care status and the cost effects of the network. Numerically, patients without pre-existing care needs had somewhat higher absolute mean 90-day costs, reflecting greater potential for intensive acute and rehabilitative interventions, whereas relative cost changes between periods were comparable between groups.

The resulting average costs were adjusted to the year of the health economics analysis (2021) using a cumulative inflation rate of 3.7% for the period 2018–2021 (24). Inflation-adjusted mean direct medical costs amounted to €11,554 in the post-implementation period group and €11,877 in the intervention group within ANNOTeM network hospitals. Direct non-medical costs were much lower at €253 and €264, respectively. Indirect costs were €117 during the pre-implementation and €98 during the post-implementation period. The total mean costs per patient within 90 days were €11,938 (95% CI: €11,280–€12,665) during the pre-implementation period and €12,252 (95% CI: €11,659–€12,836) during the post-implementation period, resulting in incremental costs of €314 per insured person (Table 2).

**Table 2:** Mean 90-day total costs per patient before and after network implementation in ANNOTeM network vs. non-network hospitals.

Hospital group	Pre-implementation period (€)	Post-implementation period (€)	Absolute Δ (€)	Relative Δ (%)
ANNOTeM network hospitals	12,252.12	12,566.44	314.32	2.6
Non-network hospitals	13,077.88	13,341.53	263.65	2.0

Mean total costs per patient (inflation-adjusted euros) within 90 days of index admission, shown for ANNOTeM network hospitals and non-network hospitals during the pre-implementation and post-implementation periods of the teleneurology network. Absolute and relative cost increases are presented for both groups.

In non-network hospitals, mean total costs per insured person increased from €13,078 (95% CI: €12,389–€13,875) during the pre-implementation period to €13,342 (95% CI: €12,640–€14,140) during the post-implementation period. Accordingly, the costs during the pre-implementation period in the non-network hospitals increased by an average of €264 per insured patient during the project period (Table 2).

In ANNOTeM network hospitals, subgroup analyses by age group showed the highest average costs among patients aged 75–84 years in both periods. Across all age categories, costs were numerically but not significantly higher in the post- compared to the pre-implementation period. The age-stratified cost data are shown in Table 3.

**Table 3:** Key cost data per age group (90 days after index admission, network hospitals).

Age group	*n*	Mean costs post-implementation period (€)	*n*	Mean costs intervention (€)
<65 years	392	12.502	233	12.576
65–74 years	358	13.067	204	13.371
75–84 years	835	13.250	448	13.425
85+ years	528	10.408	388	10.793

Mean total 90-day costs per patient by age group and study phase in ANNOTeM hospitals. “*n*” indicates patients per subgroup. All amounts in inflation-adjusted euros.

In ANNOTeM network hospitals, the mean total costs per patient within 90 days after index admission were €11,938 in the pre-implementation period and €12,252 in the post-implementation period. Following network implementation, direct medical costs increased from €11,554 to €11,877, direct non-medical costs from €253 to €264, and indirect costs decreased from €117 to €98 per patient (Table 4).

**Table 4:** Cost overview by category (90 days after index admission, network hospitals).

Cost category	Post-implementation period (€)	Intervention (€)
Direct medical costs	11.554	11.877
Direct non-medical costs	253	264
Indirect costs	117	98
Total costs	11.938	12.252

Main 90-day cost components per patient in ANNOTeM hospitals, by study phase. Values include mean direct medical, non-medical, indirect, and total costs (inflation-adjusted euros).

The availability of complete statutory health insurance claims data; cases with missing or incomplete records (such as insurer changes or unavailable datasets) were excluded from the analysis. Further, all patients insured by AOK Sachsen-Anhalt were excluded as cost data were not available for this group. Thus, of 7,640 patients included in the outcome analysis, only 7,302 were available for the present cost analysis. Of the 7,302 patients, 2,113 were in the pre-implementation and 1,273 in the post-implementation period of ANNOTeM network hospitals, 2,135 were in the pre-implementation and 1,781 in the post-implementation period of non-network hospitals.

Sensitivity analyses, including data provided for patients admitted during the COVID-19 period, confirmed the robustness of the primary findings across both the network and non-network hospitals. Specifically, in non-network hospitals, the mean total cost per patient with an acute neurological diagnosis increased from €13,077.88 (95% CI: €12,389.06–13,874.90) before the pandemic to €13,341.53 (95% CI: €12,639.99–14,139.28) during the project period, which included the COVID-19 waves. This represents a cost increase of €263.65 per case. In network hospitals, the telemedical intervention resulted in an incremental cost of €314.32 per case during the pandemic period when increased disease severity and longer treatment durations were observed.

Despite these increases, the proportional distribution of costs among direct medical treatments (inpatient, rehabilitation, outpatient, pharmaceuticals), non-medical services, and indirect costs did not change substantially between pandemic and non-pandemic phases, highlighting the robustness of the underlying cost structure.

## 4 Discussion

This study provides a comprehensive analysis of the cost-effectiveness of a quality-managed teleneurology network for rural hospitals, resulting in a modest non-significant cost increase in per-patient costs. The health-economic evaluation presented here complements the previously published outcome study by Erdur et al., which demonstrated improved 90-day clinical outcomes in the same ANNOTeM population, by quantifying associated short-term costs and deriving incremental cost-effectiveness estimates (21). In ANNOTeM network hospitals, inclusion of the €715.33 per-case intervention surcharge yielded mean 90-day costs of €12,252.12 vs. €11,937.81 in the pre-implementation period (incremental costs €314.32 per insured person), whereas structurally similar non-network hospitals showed higher absolute costs and a comparable increase (from €13,077.88 to €13,341.53; +€263.65 per case). Taken together with the improved clinical outcomes, this pattern suggests that the teleneurology network achieves better results at only modest additional per-patient costs and remains economically competitive relative to routine care in non-network hospitals.

Importantly, a similar non-significant increase occurred in non-network hospitals, suggesting that overall cost trends reflect broader developments in health care utilization and prices rather than the network itself. Indeed, the average annual increase in overall health care expenditure in the German health system between 2018 and 2021 was 8.8% (25) reflecting both price and volume effects, and therefore exceeding the cumulative general inflation adjustment of 3.7% (2018–2021) that was applied to standardize our claims-based cost estimates.

This comparison is intended to contextualize overall spending dynamics rather than to recalculate costs. Even under this conservative assumption, the incremental cost-effectiveness ratio (€14,968 per avoided adverse event) remains within ranges commonly discussed in the international health-economic literature, supporting the cost-effectiveness of the ANNOTeM network. The implementation costs for establishing the TeleNeuro-Units, which were financed outside the statutory insurance system, were not included in our claims-based estimates; amortizing these expenditures over several years would be expected to increase the ICER only marginally and is unlikely to alter the overall interpretation.

The detailed analysis of cost components indicates that most of the moderate cost increase was due to direct medical costs, which rose per patient after network implementation. This likely reflects enhanced, guideline-based acute and rehabilitative care and the costs for the 24/7 operating teleneurology service. Direct non-medical costs (such as household help and transport) remained low and stable (from €252.99 to €263.87), while indirect costs (mainly productivity losses) slightly decreased (from €116.89 to €97.82). Thus, total mean costs increased only moderately, and cost patterns were consistent across age, sex, and care-need subgroups.

These findings are consistent with other studies. While absolute cost savings reported in large US telestroke networks (e.g., $350,000 annually) (15) may not translate directly to Germany, ANNOTeM's results show that improved clinical outcomes are associated with at most modest increases in per-patient costs, resulting in favorable cost-effectiveness aligned with international experience (15). Switzer et al. showed that the Mayo Clinic's telestroke model led to increased thrombolysis rates, shorter acute stays, more discharges to home, and fewer long-term care transitions. Although the short-term incremental ICER occasionally exceeded willingness-to-pay thresholds, long-term horizons reduced the ICER to as low as $2,500 per QALY gained, well below common thresholds (14). In Europe, Kadel et al. found that Italian telemedicinenetworks for neurosurgical emergencies demonstrated per-patient savings of €1,800–2,400, mostly from avoiding unnecessary transfers without impairing clinical quality (13). Systematic reviews have confirmed that neurological telerehabilitation and telecare routinely produce per-person savings ranging from several hundred to several thousand euros, while maintaining or improving patient outcomes and satisfaction; these effects are particularly pronounced when network volume and scale are substantial (12, 26). In this context, the incremental cost per patient in ANNOTeM (€314) and the ICER of approximately €14,968 per avoided adverse event fall well within accepted thresholds.

The relatively short observation period may likely underestimate the full clinical and economic potential of the ANNOTeM network. International health-economic models consistently show that longer follow-up periods capture additional quality-adjusted life year (QALY) gains, improved functional independence, and avoidance of institutional care, leading to lower ICERs (14, 15). Maida et al. also emphasized that international studies consistently report improved ICER values when societal and post-acute effects are considered over time (26). Based on the detailed findings of Tan et al. (27), a comparison can be drawn to ANNOTeM regarding the time horizon of cost-effectiveness analyses in telestroke networks. Their results show that telestroke is already cost-effective, and in some cases cost-saving, within 90 days, driven by improved acute outcomes and reduced transfer needs.

However, in line with the accompanying ANNOTeM health economic evaluation, we did not observe statistically significant effect modification of the intervention's impact by baseline care dependency status. Patients without pre-existing care needs may derive particularly substantial long-term benefits from such networks through avoided institutionalization and maintained functional independence, which are only partially reflected in 90-day cost data.

A previous analysis of a similar network in Germany but with a follow-up of 30 months yielded slightly (but not significantly) lower costs in hospitals treating stroke patients according to the TeleStroke Unit concept with clearly lower costs for post-stroke nursing care overcompensating the higher costs for acute inpatient treatment (28).

From a health-economic perspective, a 90-day horizon and the lack of quality-of-life data are likely to underestimate the true value of the TeleNeuro Unit concept, because most cost and QALY gains arise from long-term avoidance of disability, institutionalization, and productivity loss. Several decision-analytic and Markov models have shown that ICERs for telestroke become substantially more favorable with longer horizons: Nelson et al. (14) reported that an initially high 90-day ICER decreased to roughly 2,400 USD per QALY over a lifetime, and subsequent North American and European hub-and-spoke models, as well as recent cost–utility analyses and systematic reviews, similarly found that extending the horizon to 3–5 years or lifetime typically shifts telestroke from borderline to clearly cost-effective or even cost-saving, largely due to reduced long-term dependency and institutional care. Considering this evidence, the 90-day ICER reported for ANNOTeM should be interpreted as a conservative lower-bound estimate; a multi-year model of survival, functional status, and care needs would be expected to yield more favorable cost-effectiveness ratios, in line with published long-term telestroke evaluations.

Transport costs, including inter-hospital transfers, were systematically included in the ANNOTeM cost analysis according to German claims data standards. However, if certain types of secondary transfers are not adequately coded or billed, some potential savings may remain unmeasured. International studies have highlighted the substantial cost savings associated with avoided transfers in teleneurological care, suggesting that the actual long-term cost-effectiveness of ANNOTeM may be even greater than reported. In China, Tan et al. estimated transfer-related savings of CNY 3,529 (US$512) saved per patient (27). Similarly, Whetten et al. identified avoided transfers as a key source of short-term cost savings in U.S. telestroke networks (29). Together, these findings highlight that the cost-effectiveness identified in ANNOTeM may be a conservative estimate. It is likely that the cost-effectiveness of the ANNOTeM network would appear even more favorable in long-term analyses.

Building on this, future studies should use longer observation periods beyond 90 days to capture major downstream cost drivers such as nursing home admission and other forms of institutional care, thereby enabling more realistic long-term cost estimates. Further research could compare alternative teleneurology network configurations and apply modelling approaches to project lifetime costs and quality-adjusted life years. In addition, evaluations should examine staff satisfaction and perceived workload in regional hospitals, as well as changes in inter-hospital transfer patterns, including whether such networks enable rural hospitals to manage more patients locally and reduce avoidable transfers. Prospective implementation studies in other rural and semi-urban regions would further help to assess generalizability and context-specific effects.

### 4.1 Limitations

Several limitations of this study should be acknowledged. First, the study relies on a controlled but not randomized study. Although the comparison of the results with those in non-network hospitals argues that the improved outcomes observed after the ANNOTeM network implementation was indeed intervention-related and with similar costs, we cannot exclude unobserved confounders. Second, the relatively short observation period is likely to underestimate the full scope of clinical and economic benefits attributable to the intervention, as long-term outcome improvements, such as maintained functional independence or avoided institutional care, might manifest only over extended follow-up periods (14, 15, 26). Third, only patients with complete routine billing data from AOK Nordost, BARMER, and Techniker Krankenkasse could be included in the health-economic analyses, whereas patients insured with AOK Sachsen-Anhalt were excluded because cost data were not available for this insurer. Thus, our cost and cost-effectiveness estimates are formally restricted to this subcohort. However, the broader effectiveness analysis, which also included AOK Sachsen-Anhalt, showed similar baseline characteristics and intervention effects across insurers, suggesting that substantial selection bias due to this exclusion is unlikely (21). While both populations are highly similar in baseline characteristics and outcomes, cost results are formally restricted to this subcohort. However, the consistency of outcome effects in the larger population supports the representativeness and robustness of the economic findings.

### 4.2 Conclusion

Taken together with the findings of Erdur et al. (21), which showed improved clinical outcomes in the same network and study population, the present results strengthen the evidence for the effectiveness of a quality managed teleneurology system in underserved regions. The parallel rise in expenditures in both intervention and non-network hospitals suggests that the moderate cost increase reflects broader developments in health care utilization rather than being directly attributable to the intervention. The outcome data confirm that the ANNOTeM network achieved measurable improvements in mortality, new need for nursing care, and institutionalization without additional cost increases beyond those observed in the post implementation period. Overall, the results support the cost-effectiveness and practical value of the TeleStroke Unit concept for acute neurological care, particularly in rural settings.

## Data Availability

The raw data supporting the conclusions of this article will be made available by the authors, without undue reservation.
